# ANXA1/FPR Signaling Contribute to Paclitaxel Resistance in Ovarian Cancer Through PI3K/AKT‐Associated PGC1α Regulation

**DOI:** 10.1002/cnr2.70637

**Published:** 2026-08-20

**Authors:** Zhou Li, Jiang Mingrui, Zhou Yueyang, Qi Hang, Liu Qing, Yan Xiaoyu, Yu Huimei, Xia Meihui

**Affiliations:** ^1^ Department of Obstetrics and Gynecology The First Hospital of Jilin University Changchun Jilin China; ^2^ Department of Pathophysiology, School of Basic Medical Sciences Jilin University Changchun Jilin China

**Keywords:** ANXA1, ovarian cancer, paclitaxel resistance, PI3K/AKT/PGC1α

## Abstract

**Background:**

Ovarian cancer has the highest mortality among gynecologic malignancies, and acquired resistance to paclitaxel severely limits its clinical efficacy.

**Aims:**

This study aimed to investigate whether the ANXA1/FPR axis contributes to paclitaxel resistance in ovarian cancer and to elucidate the underlying molecular mechanisms.

**Methods and Results:**

Using the ANXA1 mimetic peptide Ac2‐26 and the FPR antagonist Boc2, we provided pharmacological evidence that activation of the ANXA1/FPR axis promotes paclitaxel resistance in ovarian cancer cells. Mechanistically, ANXA1/FPR signaling activated the PI3K/AKT pathway and upregulated PGC1α, leading to enhanced mitochondrial biogenesis and adaptive energy metabolism remodeling, which in turn prevented paclitaxel‐induced cell death.

**Conclusion:**

Our findings suggest that the ANXA1/FPR/PI3K/AKT/PGC1α axis may represent a potential therapeutic target for overcoming paclitaxel resistance in ovarian cancer.

## 1 Introduction

Ovarian cancer is the most lethal gynecological malignancy [1]. Current treatment includes cytoreductive surgery combined with platinum‐based chemotherapy and paclitaxel. In recent years, neoadjuvant chemotherapy (NACT) has been increasingly used for patients with extensive disease, and emerging strategies such as PARP inhibitors, anti‐angiogenic agents, and immune checkpoint inhibitors have shown clinical benefits [2, 3, 4]. However, chemoresistance remains a major obstacle, leading to relapse and poor prognosis. Resistance can be intrinsic or acquired, involving mechanisms like enhanced drug efflux, apoptosis evasion, and metabolic reprogramming [5]. Paclitaxel resistance, in particular, severely limits efficacy [6]. Increasing evidence showed the PI3K/AKT/mTOR signaling pathway may play key roles in cancer proliferation and survival. Studies have suggested that its aberrant activation was involved in platinum and paclitaxel resistant mechanism [7].

It has been reported that Annexin A1 (ANXA1) can induce AKT phosphorylation by activating PI3K [8]. ANXA1 is a kind of calcium‐phospholipid binding protein that interacts with formylpeptide receptor (FPR1/FPR2) and participates in many important biological processes, such as cell signal transduction, differentiation and apoptosis. ANXA1 expression is tissue‐specific and varies in different tumors [9]. Studies have found that high ANXA1 expression is associated with poor chemotherapy efficacy and poor prognosis in patients with rectal cancer [10]. In contrast, silencing ANXA1 with specific inhibitors increased cell sensitivity to Cisplatin treatment [11]. Recently AC2‐26 [12] and Boc2 [13] have been found to be used as mimetic peptide of Annexin and inhibitor of ANXA1 through regulating FPR [14]. Phosphoinositol 3‐kinase (PI3K) consists of p85 regulatory subunit and p110 catalytic subunit. Our previous findings have suggested ANXA1 and PIK3R1 were highly expressed in paclitaxel‐resistant ovarian cancer cell lines. However, the role of ANXA1 in ovarian cancer chemotherapy resistance needs to be further explored.

Transcription factor Peroxisome Proliferative Activated Receptor, Gamma, Coactivator 1 alpha (PGC1α) is critical molecules in mitochondrial biogenesis and energy metabolism. Studies found upregulated PGC1α could increase the activity of antioxidant enzymes, which suppressed the apoptosis induced by excessive ROS production and promoted cancer cells migration [15]. Currently, PGC1α has also been shown to be associated with cisplatin resistance in ovarian cancer [16]. Furthermore, PGC1α may activate a variety of transcription factors including NRF1 (nuclear respiratory factor 1) and NRF2 (nuclear respiratory factor 2), which regulate mitochondrial respiratory chain proteins synthesis through mediating nuclear‐encoded mitochondrial proteins, as well as mtDNA transcription factors A (TFAM) [17, 18]. These proteins maintain cell survival by coordinating the complex regulatory network of mitochondrial biogenesis.

This study intends to explore the role of ANXA1/FPR signaling in ovarian cancer paclitaxel resistance using pharmacological tools. Our findings indicate that activation of the ANXA1/FPR axis promotes ovarian cancer cell survival, potentially through increasing mitochondrial biogenesis via the PI3K/AKT/PGC1α pathway. These results provide pharmacological evidence for the involvement of this axis and suggest that targeting ANXA1/FPR signaling may offer a new strategy for treating ovarian cancer drug resistance.

## 2 Materials and Methods

### 2.1 Cell Lines and Culture

A2780 and A2780/PA cells were cultured in RPMI‐1640 (Gibco Life Technologies, Carlsbad, California, USA) supplementing with 10% fetal bovine serum (Gibco Life Technologies, Carlsbad, California, USA), penicillin (100 U/mL), and Streptomycin (100 μg/mL). The cell culture incubator was maintained with 5% CO_2_ at 37°C. The paclitaxel‐resistant A2780/PA cell line was established by exposing parental A2780 cells to stepwise increasing concentrations of paclitaxel over approximately 6 months. The initial paclitaxel concentration was 0.066 μg/mL (approximately 1/10 of the IC50 of A2780 cells, which is 0.664 μg/mL) and was gradually increased to 40 μg/mL. During this process, the cells were periodically tested for paclitaxel sensitivity using the MTT assay to confirm the development of resistance. All cells were routinely tested for mycoplasma and confirmed negative.

### 2.2 Reagents and Antibodies

Boc2 and PKI‐402 were purchased from MCE (Monmouth Junction, New Jersey, USA). All antibodies used in this study are listed in Table 1. All other chemicals used in this study were of analytical grade and were commercially available.

**TABLE 1:** Antibodies.

Primary antibodies	Provider	Catalog no.	Dilution ratio
Annexin A1	ABclonal	A1118	1:1000
Cleaved‐ caspase 3	Proteintech	25128‐1‐AP	1:1000
BAX	Proteintech	50599‐2‐Ig	1:50 000
BCL2	Proteintech	12789‐1‐AP	1:5000
PI3K	Proteintech	20584‐1‐AP	1:1000
AKT	Proteintech	10176‐2‐AP	1:10 000
P‐AKT	Proteintech	28731‐1‐AP	1:5000
β‐Actin	Proteintech	20536‐1‐AP	1:10 000
PGC1α	Proteintech	66369‐1‐Ig	1:10 000
NRF1	Proteintech	12482‐1‐AP	1:1000
NRF2 (GABPA)	Proteintech	21542‐1‐AP	1:5000
TFAM	Proteintech	22586‐1‐AP	1:10 000
Mouse	Proteintech	SA00001‐1	1:2000
Rabbit	Proteintech	SA00001‐2	1:2000
P‐PI3K	Affinity biosciences	AF3242	1:1000

### 2.3 Peptide Sequence Synthesis

Ac2‐26, a simulated peptide of ANXA1, was synthesized by Shanghai Hanhong Science & Technology Co LTD. (Shanghai, China). The sequence information is as follows:

Ac‐Ala‐Met‐Val‐Ser‐Glu‐Phe‐Leu‐Lys‐Gln‐Ala‐Trp‐Phe‐Ile‐Glu‐Asn‐Glu‐Glu‐Gln‐Glu‐Tyr‐Val‐Gln‐Thr‐Val‐Lys‐OH.

### 2.4 MTT Assay

Cell were patched in 96‐well plate and the cell concentration was about 1.2 × 10^4^ cells/well. The cells were cultured overnight at 37°C in 5% CO2. The next day, 20 μL 5 mg/mL MTT (sigma‐aldrich, St. Louis, Missouri, USA) was added into each well which was drug treated for 24 h followed by 4 h incubation. Then the supernatant was discarded and 150 μL of DMSO was added followed by 10 min shaking. Optical density (OD) at 490 nm was measured on a microplate reader.

### 2.5 Western Blot Analysis

The treated cells were added with RIPA lysate containing protease inhibitors, fully lysed, and centrifuged at 4500 rpm/min for 15 min. Supernatant was taken, and protein concentration was detected by BCA method. The prepared protein samples were subjected to SDS‐PAGE gel electrophoresis. The isolated protein was transferred to PVDF membrane, sealed with 5% milk powder, and the primary antibody was incubated at 4°C overnight. The film was washed with TBST for three times and incubated at room temperature for 2 h with secondary antibody. The film was detected by ECL luminescence colorimeter.

### 2.6 Quantitative Real‐Time PCR

The cDNA (10 ng) was mixed with 10 μL SYBR Green Q‐PCR SuperMix (Biomake), contained 10 pmol of forward and reverse primer in a final volume of 20 μL. The q‐PCR reaction conditions were as follows: 95°C for 30 s; Denaturation: 95°C 5 s; Annealing: 50°C–60°C for 30 s; Elongation: 72°C for 10 s, in which denaturation and annealing steps repeat 30 cycles. The 2^−ΔΔCt^ relative quantitative method was used to standardize the relative expression level of each target gene through the Ct value of GAPDH for comparison between groups and mapping. The details of the primers are provided in Table 2.

**TABLE 2:** Sequence of related primers is as follows.

The name of the primer	Sequence (5′–3′)	*T*_*m*_
GAPDH	Forward: TGACTTCAACAGCGACACCCA	62.18
	Reverse: CACCCTGTTGCTGTAGCCAAA	61.09
ANXA1	Forward: GAGGAGGTTGTTTTAGCTCTGC	59.26
	Reverse: AGCAAAGCGTTCCGAAAATCT	59.12
PIK3R1	Forward: TGGACGGCGAAGTAAAGCATT	60.61
	Reverse: AGTGTGACATTGAGGGAGTCG	59.73

### 2.7 Annexin V/PI Flow Cytometry Analysis

The treated cells were collected and added to precooled PBS for washing and then suspended in 100 μL binding buffer. The negative control group was set. The experimental group was added with 2 μL annexin‐V‐FITC (20 μg/mL) and 1 μL PI (50 μg/mL) (BD, Bioscience, Franklin, Lakes, New Jersey, USA), mixed gently, and incubated at room temperature, dark, for 15 min. After incubation, 400 μL of precooled PBS was added and analyzed by flow cytometry as soon as possible. The gating strategy was as follows: viable cells were first gated on FSC‐A versus SSC‐A to exclude debris, then single cells were selected using FSC‐A versus FSC‐H. Quadrants for Annexin V‐FITC/PI staining were set using unstained cells and single‐stained control.

### 2.8 Measurement of ATP Production

Logarithmic growing cells were inoculated into six‐well plates with a cell concentration of about 3 × 10^5^ cells/well. After dosing, the cells were collected, fully lysed, centrifuged at 4°C 12 000 g for 5 min, and the supernatant was taken for subsequent determination. The control group and the experimental group were set up, 20 μL sample and 100 μL ATP detection reagent were added to each well, and the RLU value was detected by a multifunction microplate analyzer.

### 2.9 Tumor Xenografts

The C3H/SCID mice (female, 6 weeks) were provided by Charles river, Peking, China. A2780/PA cells were subcutaneously implanted into the lower flank of nude mice. Tumor volume was recorded every 3 days. The mice in the experimental group were intraperitoneally injected with Paclitaxel (5 mg/kg, NaCl 100 μL) and Boc‐MLF (20 mg/kg, NaCl 100 μL) every 3 days, and the control group was given 100 μL NaCl at the same time and in the same way. The mice would be sacrificed at Day 15.

### 2.10 Statistical Analysis

All data involved in this paper were in the form of mean ± standard deviation, and statistical analysis was conducted by SPSS 18.0 software. Two‐independent sample *t*‐test was used for comparison between two groups, and one‐way ANOVA followed by Tukey's post hoc test was used for multiple group comparisons. The quantization value of electrophoretic strip is represented by the ratio of gray value calculated by Image J software to gray value of reference.

## 3 Results

### 3.1 Highly ANXA1 Expression May Be Related With Paclitaxel Sensitivity of Ovarian Cancer Cells

**FIGURE 1:**
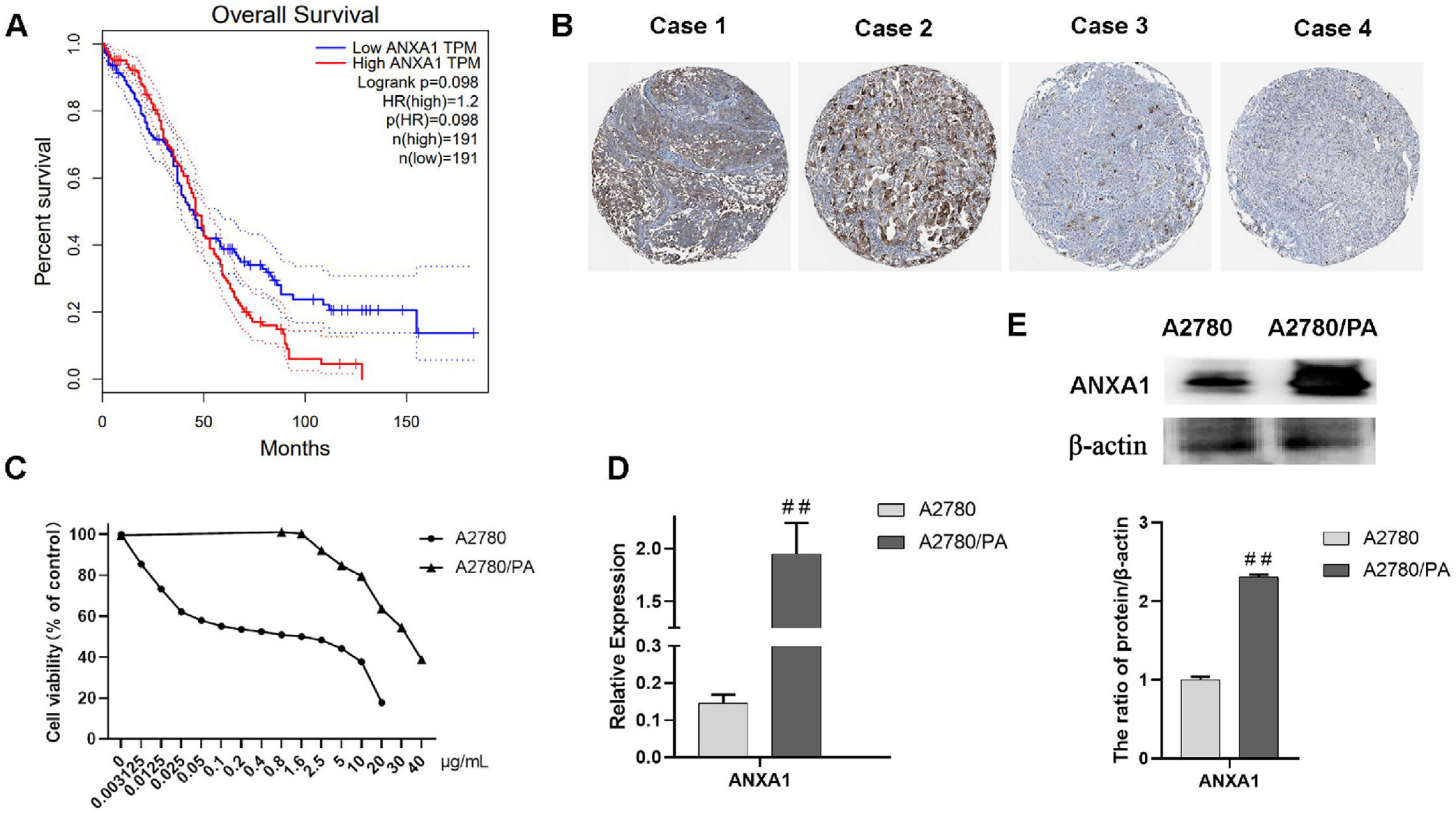
High expression of ANXA1 may be related to paclitaxel sensitivity of ovarian cancer cells. (A) The relationship between the expression of ANXA1 and the survival of ovarian cancer patients was analyzed by the TCGA database. (B) The expression of ANXA1 was analyzed by immunohistochemistry results of ovarian cancer clinical samples in The Human Protein Atlas database (40×). (C) Dose–response curves of A2780 and A2780/PA cells treated with different concentrations of paclitaxel. IC50: 0.664 μg/mL (A2780) and 31.47 μg/mL (A2780/PA). (D) ANXA1 mRNA expression of A2780 and A2780/PA was detected by qPCR assay. (E) The protein expression of ANXA1 in A2780 and A2780/PA was detected by Western blot. ^##^ *p* < 0.01 compared with A2780 group.

TCGA database was used to analyze the relationship between ANXA1 expression and survival of ovarian cancer patients, and the results showed that the higher ANXA1 expression, the shorter the lifespan (Figure 1A). Furthermore, immunohistochemistry results of ovarian cancer clinical samples from The Human Protein Atlas database showed significantly increased ANXA1 expression (Figure 1B).

To further investigate the role of ANXA1 in ovarian cancer, the sensitivity of A2780 and paclitaxel‐tolerant A2780/PA cells to paclitaxel was examined. MTT dose–response curves (Figure 1C) showed that A2780/PA cells were more resistant to paclitaxel than A2780 cells, with IC50 values of approximately 0.664 μg/mL for A2780 and 31.47 μg/mL for A2780/PA. Subsequently, the expression of ANXA1 was detected in both cell lines. The results showed that both mRNA and protein levels of ANXA1 were highly expressed in A2780/PA cells compared to A2780 cells (Figure 1D,E). These findings suggest that high expression of ANXA1 is potentially involved in the mechanism of paclitaxel resistance in ovarian cancer cells.

### 3.2 Inhibition of ANXA1 Increases the Apoptotic Sensitivity of Ovarian Cancer Cells to Paclitaxel

**FIGURE 2:**
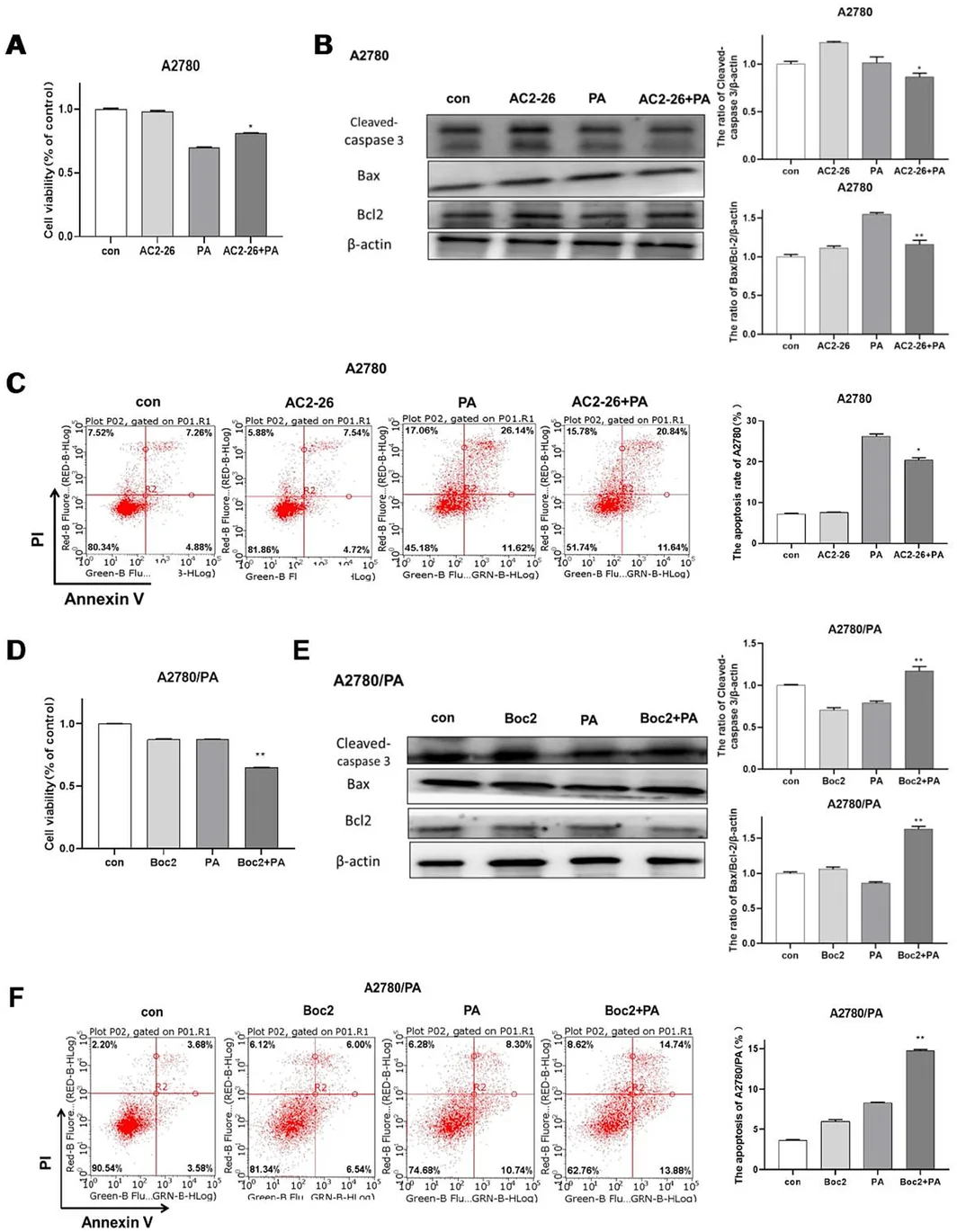
Inhibition of ANXA1 increases the apoptotic sensitivity of ovarian cancer cells to Paclitaxel. (A) The activity of A2780 cells treated with paclitaxel after the addition of AC2‐26 was detected by MTT. (B) Western Blot analysis of cleaved‐caspase3, Bax, and Bcl2 protein expression in A2780 cells treated with paclitaxel after AC2‐26 addition. In addition, quantification analysis of Cleaved‐caspase3, Bax, and Bcl2 protein and Bax/Bcl2 ratio are shown on the right. (C) The apoptosis rate of A2780 cells treated with paclitaxel after adding AC2‐26 was detected by Annexin V‐FITC/PI double staining. The right figure shows the quantitative analysis of the results. (AC2‐26:3 μm, PA: 0.00625 μg/mL, *p < 0.05 compared with PA group, **p < 0.01 compared with PA group). (D) The activity of A2780/PA cells treated with paclitaxel after the addition of Boc2 was detected by MTT. (E) Western Blot analysis of cleaved‐caspase3, Bax, and Bcl2 protein expression in A2780/PA cells treated with paclitaxel after Boc2 addition. Quantification analysis of Cleaved‐caspase3, Bax, and Bcl2 protein and Bax/Bcl2 ratio are shown on the right. (F) The apoptosis rate of A2780/PA cells treated with paclitaxel after adding Boc2 was detected by Annexin V‐FITC/PI double staining. The right figure shows the quantitative analysis of the results. (Boc2:10 μm, PA: 10 μg/mL, *p < 0.05 vs. PA group, **p < 0.01 compared with PA group).

To further verify the relationship between ANXA1 and the sensitivity of ovarian cancer cells to paclitaxel, we used the ANXA1 mimetic peptide Ac2‐26. Based on the IC50 values determined in Figure 1C, sub‐lethal concentrations of paclitaxel (0.00625 μg/mL for A2780 and 10 μg/mL for A2780/PA) were selected for the following mechanistic experiments to allow observation of pathway modulation without complete cell death. MTT results showed that Ac2‐26 increased the survival rate of A2780 cells at the same dose of paclitaxel (Figure 2A). Detection of apoptosis‐related proteins by Western Blot showed that AC2‐26 inhibited Paclitaxel‐induced Caspase3 cleavage activation and promoted the expression of anti‐apoptotic protein BCL‐2 (Figure 2B). At the same time, flow cytometry results showed that AC2‐26 could reduce the apoptosis rate of ovarian cells (Figure 2C).

Studies have indicated that ANXA1 performs biological functions by binding to formyl peptide receptor (FPR1/FPR2) [19, 20]. Boc2 [*N*‐tert‐butoxycarbonyl‐l‐Phe‐d‐Leu‐l‐Phe‐d‐Leu‐l‐Phe] is a total antagonist of formyl peptide receptor. MTT results showed that Boc2 combined with Paclitaxel significantly reduced the survival rate of A2780/PA cells (Figure 2D). Correspondingly, flow cytometry and WB results showed that Boc2 increased Paclitaxel‐induced apoptosis of A2780/PA (Figure 2E,F). These results suggest that activation of the ANXA1/FPR axis promotes paclitaxel resistance in A2780/PA cells by inhibiting apoptosis.

### 3.3 ANXA1 Can Promote the Activation of PI3K/AKT Signaling Pathway

**FIGURE 3:**
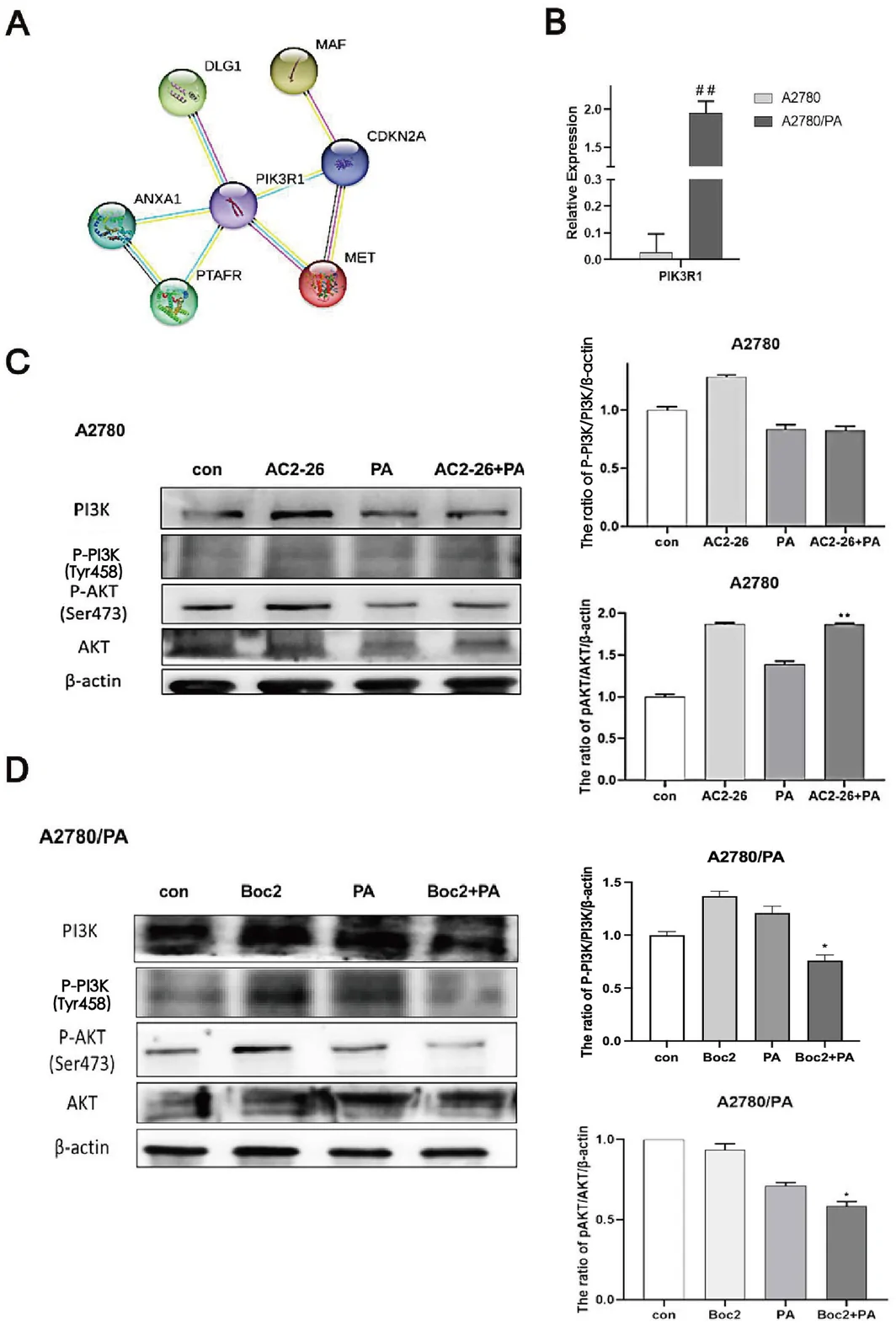
ANXA1 can promote the activation of PI3K/AKT signaling pathway. (A) The STRING database was used to predict ANXA1 related proteins. (B) The mRNA expression of PIK3R1 in A2780 and A2780/PA was detected by qPCR assay, ^##^ *p* <0.01vs.A2780 group. (C) The protein expressions of PI3K, P‐PI3K, P‐Akt and AKT in A2780 cells treated with paclitaxel after AC2‐26 addition was detected by Western Blot. The figure on the right shows the quantitative analysis of protein expression as P‐Akt/AKT ratio. (AC2‐26:3 μm, PA: 0.00625 μg/mL, **p < 0.01 compared with PA group). (D) The protein expressions of PI3K, P‐PI3K, P‐Akt, and AKT in A2780/PA cells treated with paclitaxel were detected by Western Blot. The figure on the right is the quantitative analysis of protein expression and P‐Akt/AKT ratio. (Boc2:10 μm, PA: 10 μg/mL, *p < 0.05 vs. PA group, **p < 0.01 vs. PA group).

We used STRING database to predict anxa1‐related proteins and found that PIK3R1, which encodes PI3K P85α subunit, may interact with ANXA1 (Figure 3A). Subsequently, further comparison of PIK3R1 mRNA expression between the two cells showed that A2780/PA cells had high expression of PIK3R1 (Figure 3B). Current studies suggest that PI3K is activated by binding to receptors located in the plasma membrane and generates PIP3 through phosphorylation of PIP2. AKT binds to PIP3 through the PH domain to form phosphorylated AKT (P‐AKT), thus initiating the PI3K/AKT signaling pathway [21, 22]. Therefore, the p‐AKT/AKT ratio represents the activation level of the PI3K/AKT signaling pathway. Our results showed that Paclitaxel inhibited the phosphorylation of AKT. However, AC2‐26 increased the p‐AKT/AKT ratio (Figure 3C). Conversely, inhibition of ANXA1 by Boc2 results in a further decrease in the p‐AKT/AKT ratio (Figure 3D). These results suggest that the high expression of ANXA1 in paclitaxel‐resistant strains may promote the activation of the PI3K/AKT signaling pathway.

### 3.4 ANXA1 Can Promote PGC1α‐Dependent Mitochondrial Biogenesis

**FIGURE 4:**
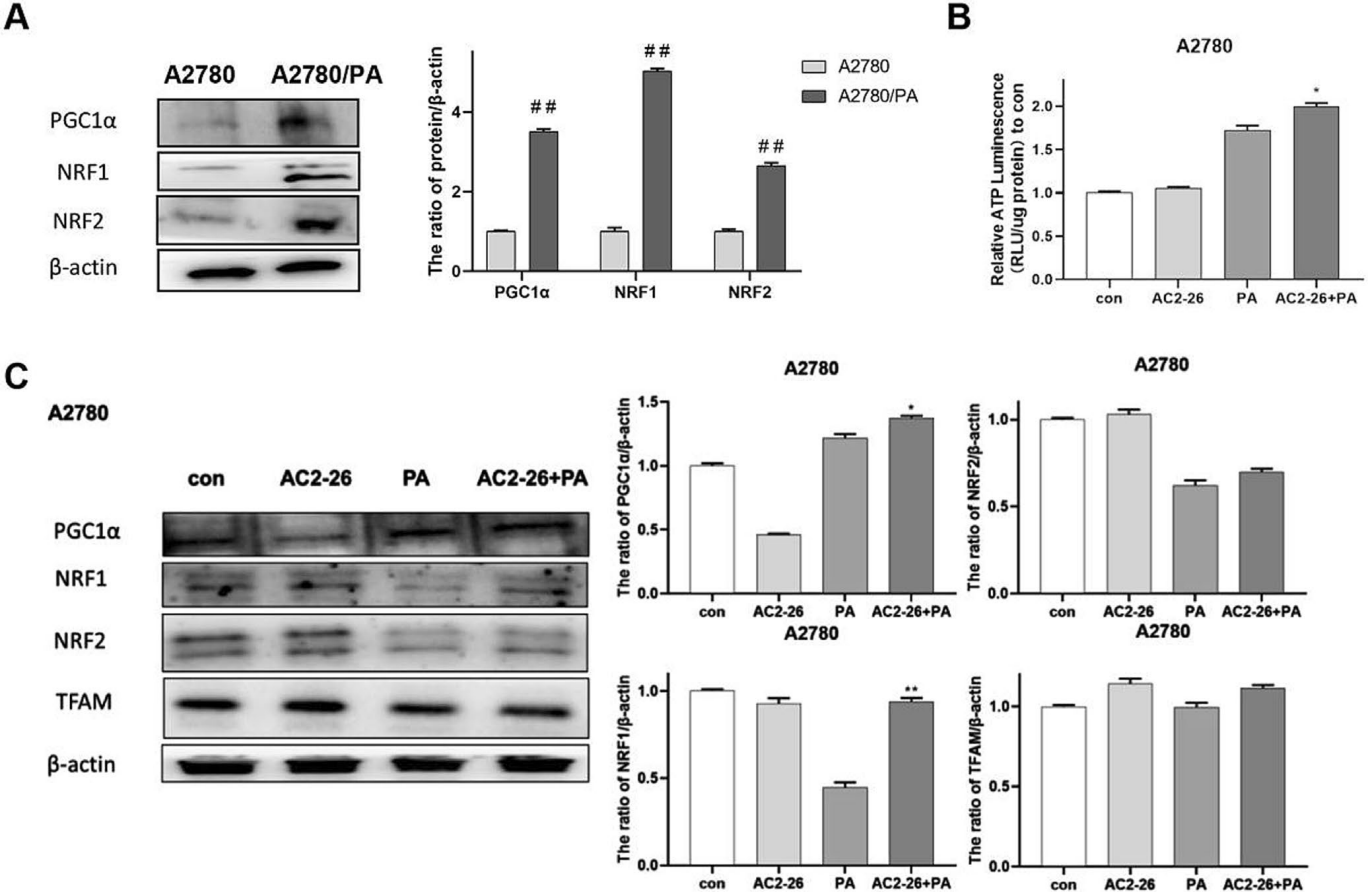
ANXA1 can promote PGC1α‐dependent mitochondrial biogenesis. (A) The protein expression levels of PGC1α and downstream related proteins NRF1 and NRF2 in A2780 cells and A2780/PA cells，^##^ *p*<0.01campared with A2780 group. The quantitative analysis was shown on the right. (B) The detection of the effect of AC2‐26 on ATP production in A2780 cells treated with paclitaxel. (C) Protein expressions of PGC1α, NRF1, NRF2, and TFAM in A2780 cells treated with paclitaxel were detected by Western Blot. Quantification analysis of protein expression was shown in the right. (AC2‐26:3 μm, PA: 0.00625 μg/mL, *p < 0.05 compared with PA group, **p < 0.01 compared with PA group).

PGC1α is a key regulator of mitochondrial biogenesis and energy metabolism, and co‐regulates mitochondrial biogenesis with downstream molecular signaling proteins NRF1, NRF2, and TFAM [23, 24]. WB results showed that compared with A2780 cells, PGC1α, NRF1, and NRF2 were highly expressed in A780/AP cells (Figure 4A). Detection of cellular ATP levels revealed that AC2‐26 treatment increased ATP production (Figure 4B). The addition of AC2‐26 increased the expressions of PGC1α, NRF1, and NRF2 to varying degrees, but is rendered inoperative to TFAM (Figure 4C). These results suggest that ANXA1 may up‐regulate mitochondrial biogenesis in A2780 cells.

### 3.5 ANXA1 May Be Involved in the Regulation of Mitochondrial Biogenesis by Up‐Regulating the PI3K/AKT Pathway

**FIGURE 5:**
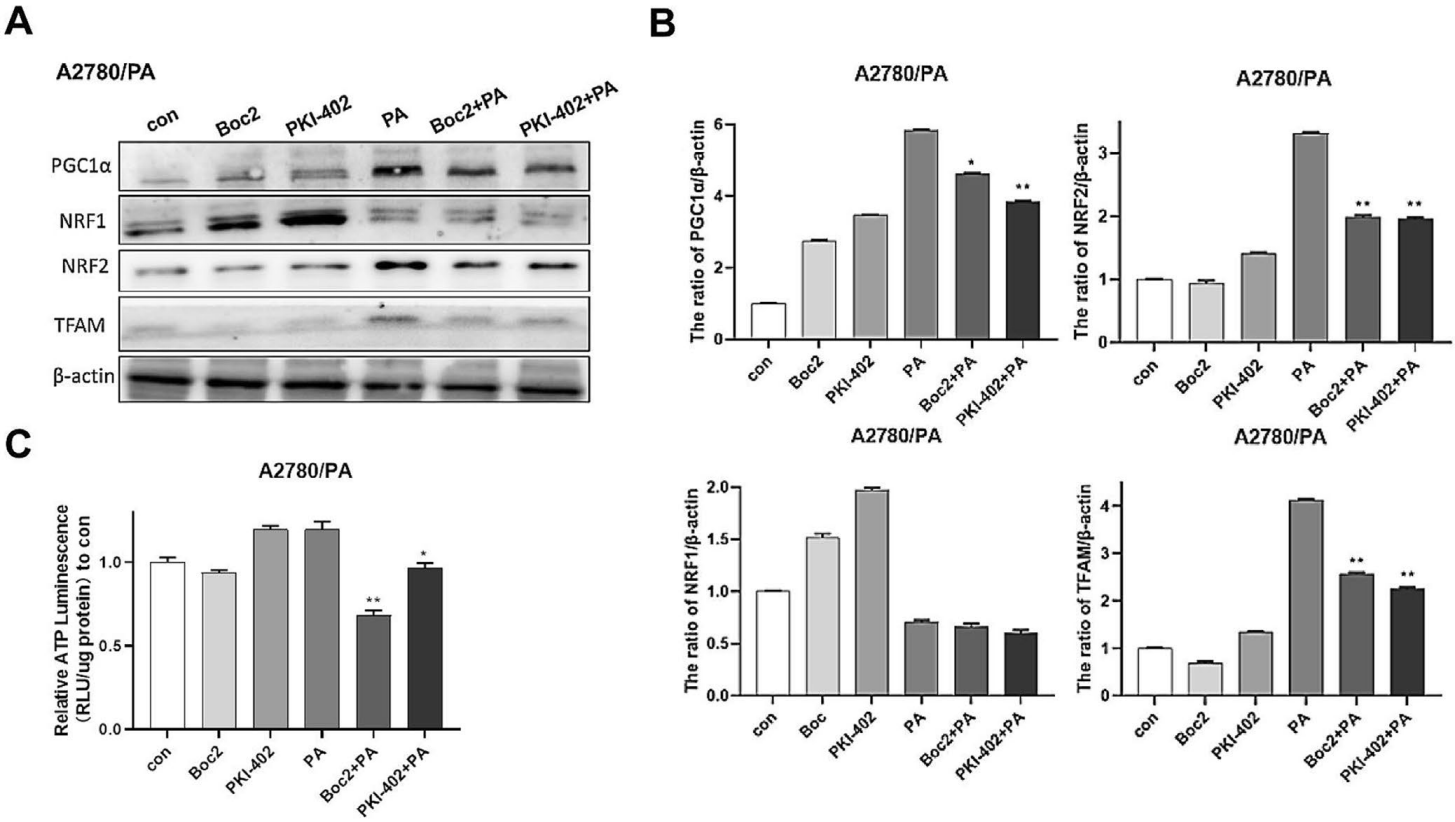
ANXA1 may be involved in the regulation of mitochondrial biogenesis by up‐regulating the PI3K/AKT pathway. (A) The protein expressions of PGC1α, NRF1, NRF2, and TFAM in A2780/PA cells were detected by Western Blot after the addition of Boc2 or PKI‐402 to paclitaxel. (B) Quantitative analysis of protein expression. (C) The detection the effect of Boc2 or PKI‐402 on ATP production in A2780/PA cells treated with paclitaxel. (Boc2:10 μm, PKI‐402:10 μm, PA:10 μg/mL, *p < 0.05 compared with PA group, **p < 0.01 compared with PA group).

To further analyze the mechanism by which ANXA1 up‐regulates PGC1α, we added Boc2, an inhibitor of ANXA1, into A780/PA cells. WB results showed that compared with the paclitaxel alone group, the combination of Boc2 and PA could inhibit the protein expression of PGC1α and downstream NRF2 and TFAM, but had no significant effect on NRF1 (Figure 5A). PKI‐402 is an effective PI3K inhibitor, which can competitively bind with the PI3K receptor ATP and inhibit the activation of the PI3K/AKT signaling pathway [25, 26]. Therefore, to verify whether ANXA1 up‐regulates mitochondrial biogenesis in A2780/PA cells through PI3K/AKT signaling, we examined the changes in mitochondrial biogenesis in A780/PA cells after inhibition of PI3K/AKT signaling. The results showed that PKI‐402 combined with PA could reduce the expression of PGC1α and downstream NRF2 and TFAM (Figure 5B). Correspondingly, ATP production was also reduced in the combination group (Figure 5C). These results together suggest that the regulatory effect of ANXA1 on PGC1α signaling may be related to the PI3K/AKT pathway.

### 3.6 Inhibition of ANXA1/FPR Signaling Suppresses the Growth of Ovarian Cancer Cells In Vivo

**FIGURE 6:**
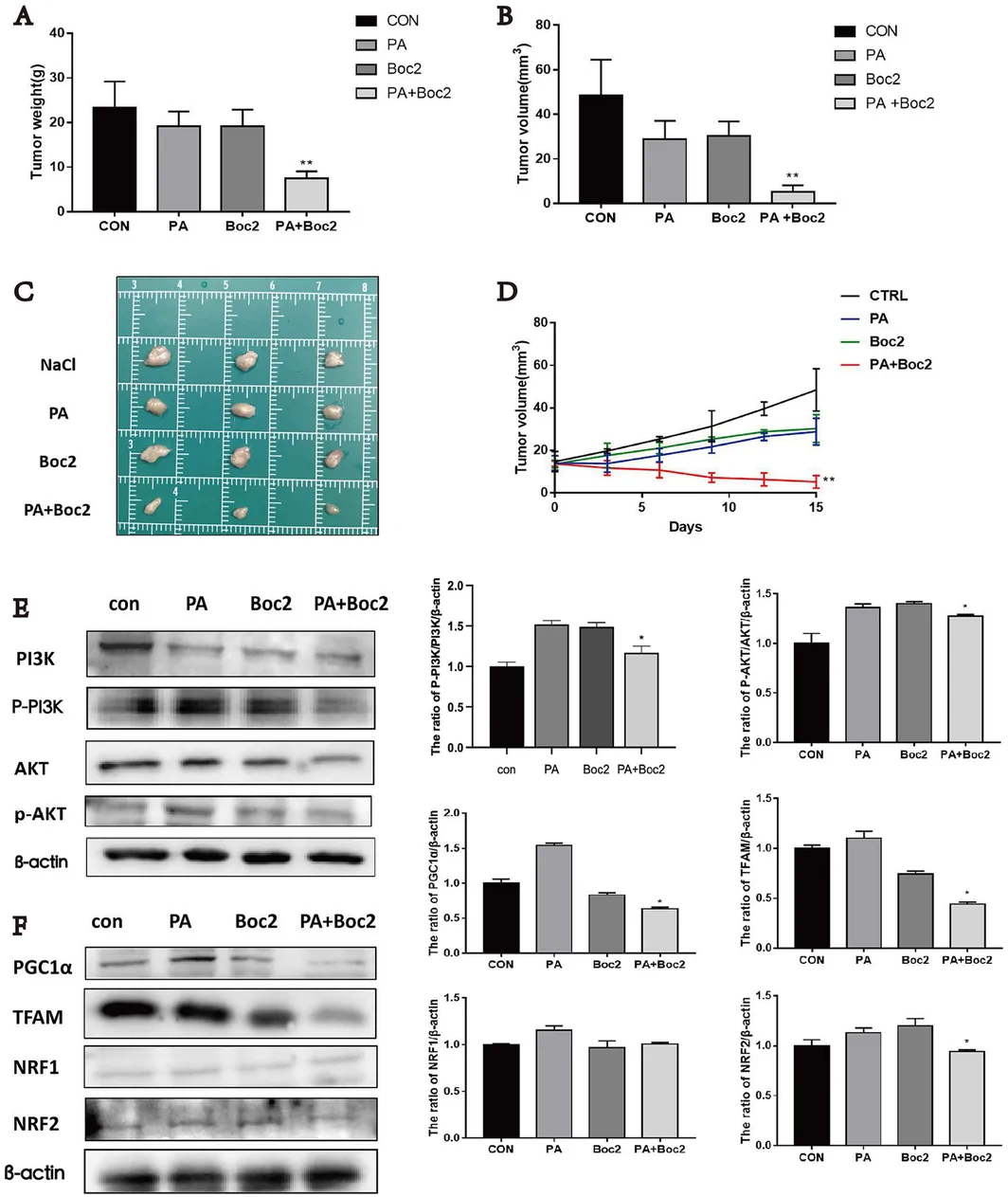
Inhibition of ANXA1/FPR signaling suppresses the growth of ovarian cancer cells in vivo. C3H SCID female mice (6 weeks old; 3 per group) were inoculated subcutaneously with A2780/PA cells (3 × 106 per animal). (A–C) Tumor weight and volumes and images of excised tumors from C3H SCID mice at 15 days. (D) Tumor volumes of excised tumors from C3H SCID mice from 0 to 15 days. (E, F) The protein expressions of PI3K, P‐PI3K, AKT, p‐AKT, PGC1α, NRF1, NRF2, and TFAM in Tumor tissues were detected by Western Blot. The quantitative analysis was shown on the right. Tumor volumes were measured as indicated. Data points are presented as mean volume ± s.d. *p < 0.05. **p < 0.01.

To better understand the role of ANXA1 in ovarian cancer development in vivo, paclitaxel‐resistant cells A2780/PA were inoculated subcutaneously into C3H SCID mice for tumor xenografts. The results showed that tumor growth was slowed after Boc2 treatment, and the combination with paclitaxel significantly inhibited tumor growth (Figure 6A). After Boc2 treatment, the final tumor volume also decreased significantly (Figure 6B–D). In conclusion, we found that pharmacological inhibition of FPR signaling significantly suppressed the growth of A2780/PA cell‐derived tumors in vivo. Targeted inhibition of FPR signaling enhanced the sensitivity of ovarian cancer cells to paclitaxel and inhibit the progression of ovarian cancer. Western blot results showed that Boc2 treatment reduced the ratio of p‐PI3K/PI3K and p‐AKT/AKT (Figure 6E). Compared with the paclitaxel mono‐plus group, the combination of Boc2 and PA could inhibit the protein expression of PGC1α and downstream NRF2 and TFAM, but had no significant effect on NRF1 (Figure 6F). Together, the results presented above demonstrate that pharmacological blockade of the ANXA1/FPR axis reduces PI3K/AKT activation, downregulates PGC1α signaling, and enhances the sensitivity of ovarian cancer cells to paclitaxel.

## 4 Discussion

Of all gynecological malignancies, ovarian cancer is the most lethal disease [27]. The standard treatment for ovarian cancer is cytoreductive surgery combined with chemotherapy [28]. Paclitaxel, originally isolated from taxus brevifolium, has been recommended as a first‐line chemotherapy agent for many cancers, including ovarian cancer [29], but drug resistance has become a major factor limiting patient efficacy. Acquired resistance to Paclitaxel in ovarian cancer patients results in a 5‐year survival rate of about 20%–30%, and more than 80% of patients with advanced ovarian cancer relapse within 3 years after initial treatment [30]. The key molecules or mechanisms related to chemical resistance in ovarian cancer are still unknown. Insensitivity to chemotherapeutic drugs is one of the main obstacles to the treatment of tumor recurrence, and overcoming Paclitaxel resistance is an important goal in the treatment of ovarian cancer. Exploring key molecules related to drug sensitivity is an essential method to reverse chemical resistance in cancer, but the key molecules or mechanisms related to chemical resistance in ovarian cancer remain to be elucidated. In this study, ANXA1 may induce Paclitaxel resistance in ovarian cancer through the PI3K/AKT/PGC1α axis, and may be used as a molecular target to reverse Paclitaxel resistance in ovarian cancer in combination with chemotherapy drugs to improve patient survival.

ANXA1 expression is tissue‐specific and varies in different tumors [8], and has been reported to be associated with chemotherapy resistance in a variety of tumors [9]. Studies have shown that Cisplatin‐resistant breast cancer cells have high expression of ANXA1, and silencing ANXA1 with specific targeted compounds increases its sensitivity to Cisplatin treatment [11]. Studies have found that high ANXA1 expression is associated with poor chemotherapy efficacy and poor prognosis in patients with rectal cancer [31]. Treatment response and prognosis of patients with esophageal squamous cell carcinoma have a linear inverse relationship with ANXA1 expression [32], and clinical studies have proved that high ANXA1 expression in breast cancer patients is associated with poor drug response to trastuzumab [33]. In addition, the mRNA level of 5‐fluorouracil‐resistant colon cancer ANXA1 cells increased 5.5 times compared to their parents, and the high expression of ANXA1 led to increased drug resistance of the cells. Conversely, the sensitivity of 5‐fluorouracil‐resistant colon cancer 5‐fluorouracil‐resistant cells to 5‐fluorouracil increased after ANXA1 knockout [34]. These results suggest that ANXA1 high expression plays a key role in the development of chemotherapeutic drug resistance, suggesting that interference with ANXA1 expression may reverse drug resistance and make ANXA1 a target for evaluating drug treatment efficacy. However, the specific mechanisms by which ANXA1 affects chemotherapeutic drug resistance remain unclear and need to be further explored. Previous studies have found that ANXA1 is highly expressed in Paclitaxel‐resistant ovarian cancer cells using SKOV3 and Paclitaxel‐resistant ovarian cancer SKOV3/PA cell lines [35]. Similarly, this study was based on ovarian cancer cell A2780 (0.664 μg/mL) and Paclitaxel‐resistant ovarian cancer cell A2780/PA (31.47 μg/mL). Both mRNA and protein levels of ANXA1 were significantly increased in A2780/PA cells.

To investigate the functional relevance of ANXA1 expression levels, we used the ANXA1 mimetic peptide Ac2‐26 and the FPR antagonist Boc2. Ac2‐26 [12] and Boc2 [13] have been mentioned in several literatures as synthetic drugs that promote and inhibit ANXA1 function, both of which act in combination with FPR [14]. These pharmacological tools allowed us to assess the impact of the ANXA1/FPR pathway modulation on paclitaxel sensitivity. Apoptosis induction of ovarian cancer cells is one of the important mechanisms by which Paclitaxel and other chemotherapy drugs play a killing role. Therefore, we evaluated the changes of Paclitaxel tolerance by observing cell activity and apoptosis level. Compared with Paclitaxel alone, the apoptosis rate was higher in the ac2‐26 combined with the Paclitaxel group, Cleaved Caspase 3 expression was decreased, and the ratio of the pro‐apoptotic protein Bax to the anti‐apoptotic protein Bcl2 was decreased, indicating decreased apoptosis level. However, in A2780/PA cells, the apoptosis rate of Boc2 combined with Paclitaxel was lower, Cleaved Caspase 3 expression was increased, and the Bax/Bcl2 ratio was increased, indicating that A2780/PA cells were in a “mitochondrial preexcitation state” or “dying state” after ANXA1 inhibition. The apoptosis rate can reflect the sensitivity and tolerance of cells to drugs; high apoptosis rate indicates high sensitivity and low drug resistance. Therefore, these results indicate that ovarian cancer cells with high ANXA1 expression are less sensitive to Paclitaxel and more resistant to it.

Studies have shown that inhibition of the PI3K/AKT signaling pathway can inhibit the proliferation of HCC cells and increase their sensitivity to Paclitaxel at the same time, suggesting that the PI3K/AKT signaling pathway may serve as a target for reversing Paclitaxel resistance. It has been reported that ANXA1 can be used as a prognostic molecular marker for trastuzumab therapy in breast cancer, and high expression of ANXA1 can lead to chemotherapy resistance through activation of PI3K/AKT, in which ANXA1 plays an essential role [36]. Therefore, we hypothesized that ANXA1 affects Paclitaxel resistance in ovarian cancer through the PI3K/AKT signaling pathway. The activation of the PI3K/AKT signaling pathway was observed by the p‐AKT/AKT ratio. It was found that the addition of ANXA1 analog peptide AC2‐26 in A2780 cells increased the p‐AKT/AKT ratio, and the addition of the FPR inhibitor Boc2 in A2780/PA cells increased the p‐AKT/AKT ratio. The expression of PI3K protein and the p‐AKT/AKT ratio were decreased. These results indicate that ANXA1 can activate the PI3K/AKT signaling pathway.

Recent studies have further confirmed the critical role of the PI3K/AKT signaling axis in ovarian cancer progression and chemoresistance. Tyagi et al. demonstrated that PKCι drives glycolysis and tumor progression in high‐grade serous ovarian cancer (HGSOC) by activating the PI3K/AKT/mTOR pathway, highlighting the central function of this pathway in the metabolic reprogramming of ovarian cancer cells [37]. Agrawal et al. comprehensively reviewed that the PI3K/AKT/mTOR pathway is a major driver of chemotherapy resistance in ovarian cancer and summarized a series of ongoing clinical trials targeting this pathway, supporting PI3K/AKT as a core actionable target for overcoming drug resistance [38].

Of note, although PIK3CA is clinically more relevant in ovarian cancer, our STRING database analysis predicted PIK3R1 (encoding the p85α regulatory subunit) as a potential interactor of ANXA1. Therefore, we focused on PIK3R1 in this study. We do not exclude that ANXA1 may also interact with PIK3CA, and further studies are needed to compare the roles of different PI3K subunits in ANXA1‐mediated paclitaxel resistance. Of course, we cannot rule out that ANXA1 has other functions besides regulating the PI3K pathway.

Previous laboratory studies have found that PGC1α expression can be increased in Cisplatin‐resistant ovarian cancer SKOV3/DDP cells, accompanied by an increase in mitochondrial mass, maintenance of mitochondrial membrane potential, and increased oxygen consumption, suggesting that PGC1α plays an important role in maintaining mitochondrial function and resistance to Cisplatin toxicity [39]. We also observed increased expression of PGC1α and related downstream proteins in Paclitaxel‐resistant OVARIAN cancer A2780/PA cells. To determine whether ANXA1 promotes the activation of the PGC1α mitochondrial biogenesis pathway, we detected the expressions of related proteins NRF1, NRF2, and TFAM. In our results, the expression of PGC1α and downstream related proteins in A2780 cells increased with the addition of the ANXA1 simulated peptide AC2‐26 in the presence of Paclitaxel, while the expression of PGC1α and downstream related proteins decreased with the addition of FPR inhibitor Boc2 in A2780/PA cells. These results indicate that ANXA1 can promote the activation of the PGC1α mitochondrial biogenesis pathway. However, the mechanisms by which PGC1α is activated and mitochondrial biogenesis is upregulated are poorly understood. Recently, it has been found that activation of the PI3K/AKT signaling pathway up‐regulates PGC1α [40]. In order to clarify whether it is affected by PI3K, we observed decreased expression of PGC1α and downstream related proteins after adding the PI3K inhibitor PKI‐402 into A2780/PA cells. Therefore, the possible mechanism is as follows: ANXA1 promotes the expression of PGC1α by activating the PI3K/AKT signaling pathway and activates the mitochondrial biogenesis pathway, thereby affecting Paclitaxel resistance in A2780/PA cells.

We acknowledge several limitations. The present study primarily relies on the ANXA1 mimetic peptide Ac2‐26 and the FPR antagonist Boc2. While these pharmacological tools provide strong evidence for the involvement of the ANXA1/FPR axis in paclitaxel resistance, they do not constitute direct genetic proof that endogenous ANXA1 is the causative factor. Ac2‐26 may have off‐target effects, and Boc2 blocks FPRs without specifically manipulating ANXA1 expression. Therefore, our findings should be interpreted as pharmacological evidence supporting a functional association rather than definitive causality. We also did not perform rescue experiments with PI3K/AKT inhibitors to directly reverse Ac2‐26‐mediated resistance; therefore, our data support association, not pathway necessity. Future studies employing ANXA1 knockdown/overexpression and rescue experiments in additional ovarian cancer models (e.g., OVCAR3, OVCAR8) are required to conclusively establish the endogenous role of ANXA1. Additionally, FPR1/FPR2 expression levels were not measured in the current study, which is another limitation.

## 5 Conclusions

In summary, our pharmacological data demonstrate that activation of the ANXA1/FPR axis promotes paclitaxel resistance in ovarian cancer cells, likely through PI3K/AKT‐dependent upregulation of PGC1α and mitochondrial biogenesis. These findings suggest that increased ANXA1 expression is associated with poor prognosis and may serve as a potential biomarker for paclitaxel resistance in ovarian cancer. However, direct genetic evidence for endogenous ANXA1 as the driver remains to be established. Nevertheless, targeting the ANXA1/FPR/PI3K/AKT/PGC1α axis may represent a promising strategy to overcome paclitaxel resistance.

## Author Contributions

**Jiang Mingrui:** software, data curation. **Qi Hang:** formal analysis, supervision. **Liu Qing:** visualization. **Xia Meihui:** writing – original draft, writing – review and editing. **Zhou Li:** conceptualization, methodology. **Zhou Yueyang:** validation, investigation. **Yan Xiaoyu:** project administration. **Yu Huimei:** resources.

## Funding

This research was funded by Jilin Provincial Research Foundation for the Development of Science and Technology Projects (YDZJ202501ZYTS052).

## Conflicts of Interest

The authors declare no conflicts of interest.

## Data Availability

The data that support the findings of this study are available from the corresponding author upon reasonable request.
